# Optimization of In Vitro Germination, Viability Tests and Storage of *Paeonia ostii* Pollen

**DOI:** 10.3390/plants12132460

**Published:** 2023-06-27

**Authors:** Mengchen Li, Fengfei Jiang, Linbo Huang, Hui Wang, Wenqing Song, Xiaoxiao Zhang, Yanlong Zhang, Lixin Niu

**Affiliations:** 1 College of Landscape Architecture and Arts, Northwest A&F University, Yangling 712100, China; limengchen@nwafu.edu.cn (M.L.); j907609866@nwafu.edu.cn (F.J.); hlb@nwafu.edu.cn (L.H.); 18339902483@nwafu.edu.cn (H.W.); songwenqing@nwafu.edu.cn (W.S.); 2 Oil Peony Engineering Technology Research Center of National Forestry Administration, Yangling 712100, China

**Keywords:** *Paeonia ostii*, optimal medium, pollen viability, unfreezing and rehydration, storage condition

## Abstract

*Paeonia ostii* is an important woody oil crop mainly cross-pollinated. However, the low yield has become an important factor restricting the industrial development of *P. ostii.* Cross-pollination has become one of the important measures to increase the seed yield. Therefore, conservation of pollen with high vitality is crucial to ensure successful pollination of *P. ostii*. In this study, we found an effective methodological system to assess the viability, ability to germinate, and optimal storage conditions of *P. ostii* pollen grains. The optimal medium in vitro was 50 g/L sucrose, 100 mg/L boric acid, 50 g/L PEG6000, 100 mg/L potassium nitrate, 300 mg/L calcium nitrate, and 200 mg/L magnesium sulfate at pH 5.4. Optimal germination condition in vitro was achieved at 25 °C for 120 min, allowing easy observation of the germination percentage and length of the pollen tubes. In addition, the viability of pollen grains was assessed by comparing nine staining methods. Among them, MTT, TTC, benzidine-H_2_O_2_, and FDA were effective to distinguish between viable and non-viable pollen, and the results of the FDA staining method were similar to the pollen germination percentage in vitro. After evaluation of pollen storage, thawing and rehydration experiments showed that thawing at 4 °C for 30 min and rehydration at 25 °C for 30 min increased the germination percentage of pollen grains stored at low temperatures. The low-temperature storage experiments showed that 4 °C was suitable for short-term storage of *P. ostii* pollen grains, while −80 °C was suitable for long-term storage. This is the first report on the in vitro germination, viability tests, and storage of *P. ostii* pollen grains, which will provide useful information for *P. ostii* germplasm conservation and artificial pollination.

## 1. Introduction

Tree peony (*Paeonia ostii*) is a perennial shrub in the family Paeoniaceae native to China. As a woody oil crop, it is widely planted in China because its seed oil contains more unsaturated fatty acids [1,2]. The yield of tree peony seeds is very important for the development of the oil peony industry [3]. However, tree peony is a kind of cross-pollination-based plant, and the production of seeds depends on the fertility of pollen, which depends on the vitality and germination of pollen of effective conservation in its production [4].

Pollen viability is crucial for cross breeding and artificial pollination. It can be determined by staining and in vitro germination. Many dyes are used to measure viability, such as methylene blue [5], acetate carmine [6], TTC [7], FDA [8], etc. The staining method is widely used in the determination of viability because of its easy operation and short time-consuming process. However, compared with the staining method, the in vitro germination method, where the growth of the pollen tube can be observed, is more accurate. Although performing germination experiments is more complicated, due to its accuracy, more and more researchers have studied different germination mediums for pollen. Brewbaker and Kwack’s (BK) medium has been widely used in germination experiments of pollen grains [9]. The medium contains different concentrations of calcium nitrate, potassium nitrate, magnesium sulfate, sucrose, and boric acid, among which sucrose and boric acid were considered necessary for pollen grains. In addition to the above substances, some culture media will add polyethylene glycol to maintain the osmotic pressure of pollen grains. Polyethylene glycol (PEG), as a polymer penetrant, has the characteristics of high viscosity and is relatively close to the physical state of the stigma surface, so it can promote germination and pollen tube growth [7,10].

Revealing the duration of pollen viability following storage under various conditions holds great importance for breeding programs, genetic preservation, and artificial pollination [11]. Artificial pollination is an important method for cross breeding and improving plant yield, and the vitality of pollen affects the success rate of artificial pollination [12]. At room temperature, due to the influence of temperature and air moisture, the viability will decrease, which is not conducive to long-term storage of pollen grains [13]. Under low-temperature conditions, pollen grains can be preserved for a long time, up to more than one year [14].

In vitro pollen germination, viability assays, and storage are the most commonly used methods in genetic improvement programs [15]. Each species requires specific germination medium and pollen viability assay protocols, as well as appropriate storage conditions to preserve germplasm [16]. Currently, viability assays, storage conditions, and in vitro germination of tree peony pollen grains have not been systematically studied. In this study, tree peony pollen grains were used to determine the germination medium, and the difference between the nine staining methods in the determination of viability was compared. At the same time, the pollen grains were stored at different temperatures to optimize the conditions for long-term storage. Our research will play a vital role in breeding efforts, germplasm conservation, and artificial pollination of tree peony.

## 2. Results

### 2.1. Optimization of In Vitro Germination Medium for Pollen

#### 2.1.1. Effects of Individual Components of Medium on Pollen Germination

**Figure 1:**
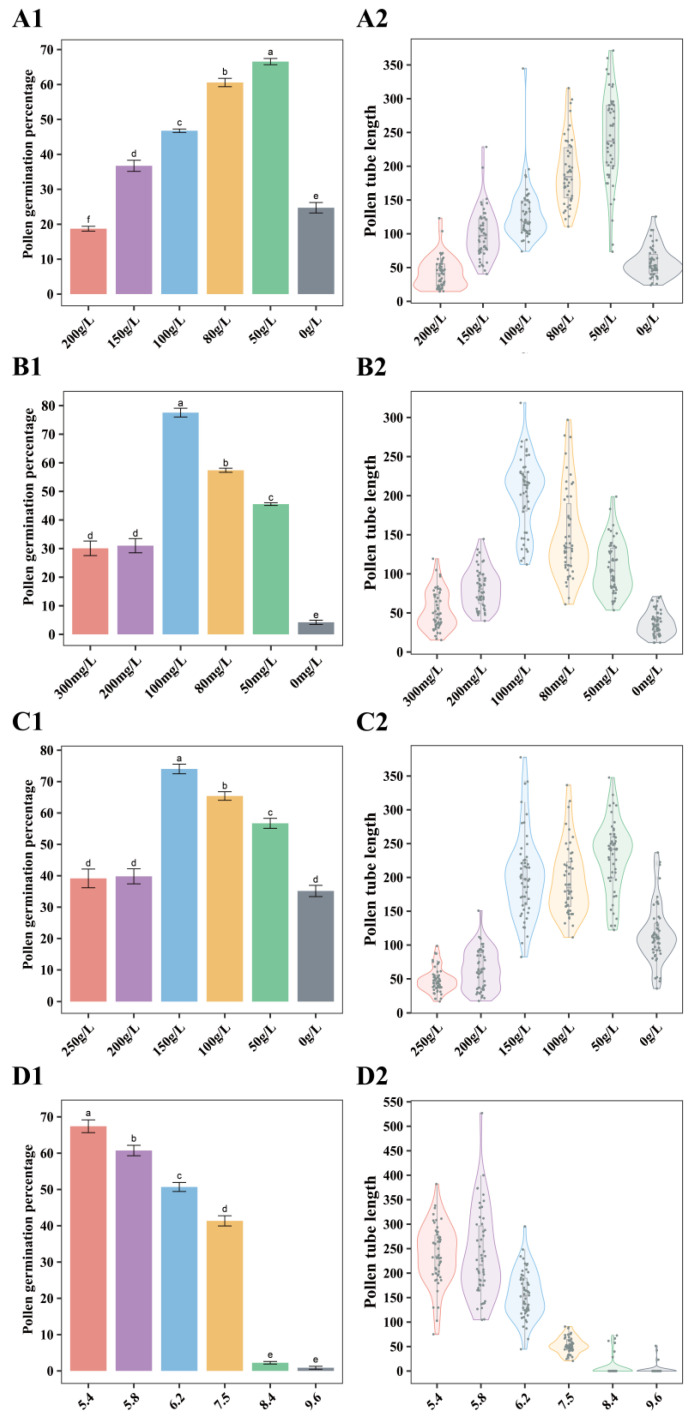
Pollen germination percentage (%) and pollen tube length (μm) of *Paeonia ostii* at different concentrations of four compositions (**A1**,**A2**): sucrose; (**B1**,**B2**): boric acid; (**C1**,**C2**): PEG6000; (**D1**,**D2**): pH. The letters represent significant differences.

In the experiment, different concentrations of sucrose (0–200 g/L) were used for germination tests. Among all tested sucrose concentrations, the germination percentage was the highest (66.55%) at 50 g/L sucrose concentration. At 200 g/L sucrose, the germination percentage was the lowest, 18.72% (Figure 1(A1)), which indicated that too high a concentration of sucrose would not promote pollen germination. When the medium did not contain sucrose, the germination percentage was only 24.72%, but the length of the pollen tube was shorter, only 58.16 μm (Figure 1(A2)).

Boric acid plays an important role in pollen germination and pollen tube growth [17]. Different concentrations of boric acid (0–300 mg/L) were tested in this experiment. Among all tested boric acid concentrations, 50 mg/L, 80 mg/L, and 100 mg/L boric acid showed higher germination percentages, which were 45.54%, 57.38%, and 77.54%, respectively (Figure 1(B1)). Among them, 100 mg/L boric acid was accompanied by the highest germination percentage. When there was no boric acid in the medium, the germination percentage was only 4.21%, and the length of the pollen tube was 36.46 μm (Figure 1(B2)).

Polyethylene glycol (PEG) has the effect of increasing osmotic pressure, preventing pollen from absorbing water and bursting during hydration [18,19]. The different concentrations of PEG (0–250 g/L) were tested. In all experiments, 50 g/L, 100 g/L, and 150 g/L PEG showed higher germination percentages, 56.71%, 65.41%, and 74.03%, respectively. Among them, 150 g/L PEG was necessary to achieve the highest germination percentage (Figure 1(C1)). With the increase or decrease of PEG concentration, the germination percentage decreased. When the medium did not contain PEG, the germination percentage was the lowest, 35.15%, and some pollen tubes swelled and burst during the growth process. When the concentration of PEG in the medium was 250 g/L, the length of the pollen tube was only 48.08 μm (Figure 1(C2)).

Pollen germination and growth of pollen tube are affected by pH, and acidic medium is suitable for germination [20]. To optimize the pH for pollen grains, we tested different values: 5.4, 5.8, 6.2, 7.5, 8.4, 9.6. In all experiments, pH 5.4, 5.8, and 6.2 showed higher germination percentages, 67.42%, 60.73%, 50.68%, respectively (Figure 1(D1)). When the pH in the medium was greater than 7, the germination percentage decreased, and even a lot of pollen grains did not grow pollen tubes (Figure 1(D2)).

The different concentrations of potassium nitrate (0–300 mg/L), calcium nitrate (0–500 mg/L) and magnesium sulfate (0–500 mg/L) were tested in germination medium. When the medium lacked potassium nitrate, calcium nitrate, and magnesium sulfate, the germination percentages were 36.89%, 39.38%, and 36.49%, respectively. The addition of potassium nitrate, calcium nitrate, and magnesium sulfate to the medium had an effect on both the germination percentage and the growth of pollen tubes. Higher pollen germination percentages were observed at 100 mg/L potassium nitrate, 300 mg/L calcium nitrate, and 200 mg/L magnesium sulfate, respectively (Figure S1).

#### 2.1.2. Optimization of In Vitro Germination Medium for Pollen

In previous experiments, it was found that different levels of sucrose, PEG, boric acid, and pH had significant effects on germination. Therefore, three levels (Table 1) of these four components were selected to optimize the germination medium, while the concentrations of calcium nitrate, magnesium sulfate, and potassium nitrate were kept at the optimal levels 300 mg/L, 200 mg/L, and 100 mg/L, respectively. The results of range analysis showed that the pH value had a significant effect on pollen germination (R = 34.34%), followed by boric acid (R = 5.72%) and sucrose (R = 4.66%). In addition, PEG6000 (R = 1.51%) had the least influence in this experiment (Table 2). The germination percentage was different in the orthogonal assay test strategy, and the germination percentage of pollen grains was between 46.63–83.36%, and the length of the pollen tube was also different (Figure S2).

As shown in Table 2, the results of orthogonal experiments showed that pollen was suitable for germination in acidic medium, and compared with other levels (H2 and H3), the 1st level of pH (H1) had the highest average value of pollen germination percentage (x1 = 82.91%). Similarly, in comparison to their 2nd (S2 and B2 in Table 2) and 3rd levels (S3 and B3), the 1st level of sucrose (S1) and PEG6000 (P1) exhibited the highest average pollen germination percentage (x1 = 71.8% and 71.64%, respectively). Moreover, boric acid had the highest average value of pollen germination percentage (x3 = 72.6%) at the 3rd level (B3) compared to their 1st (B1) and 2nd (B2) levels. The different levels for the four factors also reflect this phenomenon (the K values in the Table 2).

Therefore, the composition of the optimal medium was sucrose, 50 g/L, boric acid, 100 mg/L, PEG6000, 50 g/L, potassium nitrate, 100 mg/L, calcium nitrate, 300 mg/L, magnesium sulfate, 200 mg/L, pH 5.4. The germination percentage was determined in the optimal medium, and the average value of six repetitions was 86.36%, which indicated that the medium was suitable for *P. ostii* pollen grains.

### 2.2. Pollen Germination and Pollen Tube Growth In Vitro at Different Temperatures

The medium was placed at different temperatures (15, 20, 25, 30, 35 °C) for germination, and after 30, 60, 90, 120, and 180 min, the germination percentage and pollen tube length were measured (Table 3 and Table 4). The germination percentage of pollen grains was found to be temperature-dependent, with variations in temperatures significantly affecting the germination of pollen grains. Among them, low temperature was not suitable for germination, and the germination percentage was only 16.33% at 15 °C for 60 min. When germinated for 120 min, the germination percentage was 52.22%, which was not significantly different from that of 180 min. Similarly, high temperature was not suitable for germination. When the germination temperature was 35 °C, the germination percentage was only 24.22% in 120 min, which was not significantly different from that in 180 min. On the contrary, the germination percentage reached 85.05% at 25 °C for 120 min, which was not significantly different from that of 180 min (Table 3).

Pollen grains germination is accompanied by elongation of the pollen tube. In addition to counting the germination percentage, the pollen tube lengths at different temperatures were also measured. Different temperatures affected the length of pollen tubes. The length of the pollen tube reached 390.76 µm at 25 °C for 180 min, but the length of the pollen tube was too long, which was not conducive to the statistics of the germination percentage (Table 4). The length of the pollen tube reached 245.06 µm at 25 °C for 120 min. On the contrary, at the same germination time, the length of the pollen tube at 15 °C and 35 °C was 135.73 and 123.66 µm, respectively, which were significantly lower than those at 20 °C, 25 °C, and 30 °C. By comparing the germination percentage and pollen tube length, it was found that culturing at 25 °C for 120 min was suitable for *P. ostii* pollen grains.

### 2.3. Comparison of Pollen Vitality Staining Methods

**Figure 2:**
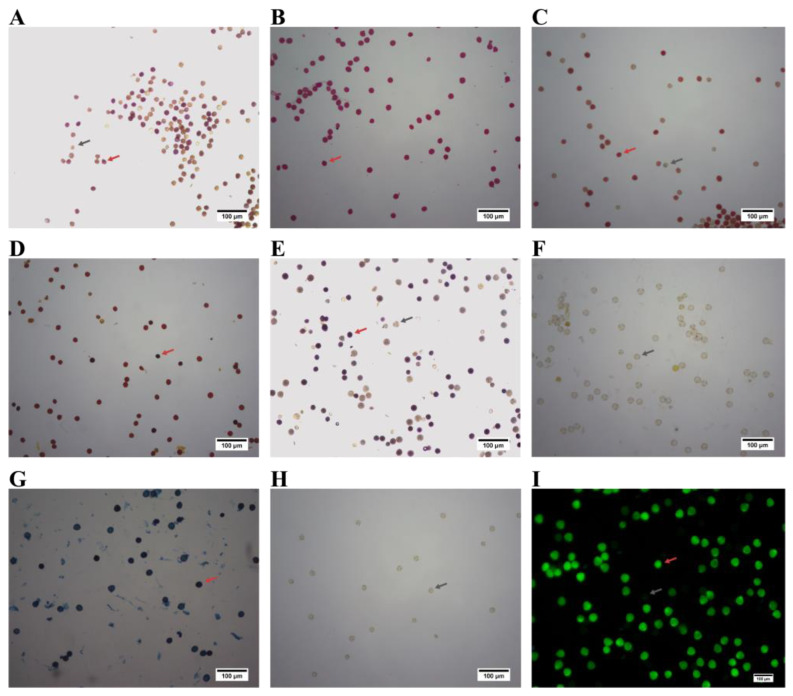
Comparison of the nine staining methods of *Paeonia ostii* pollen grains. (**A**) MTT staining method; (**B**) Acetic carmine staining method; (**C**) TTC staining method; (**D**) I_2_-KI staining method; (**E**) Benzidine-H_2_O_2_ staining method; (**F**) Peroxide staining method; (**G**) Methylene blue staining method; (**H**) Inorganic acid staining method, and (**I**) FDA staining method. Grey arrows indicate non-viable pollen; red arrows indicate viable pollen.

It was determined that the four staining methods MTT, TTC, benzidine-H_2_O_2_, FDA could identify viable and non-viable pollen. For MTT staining, viable pollen was stained red (Figure 2). In addition, for the TTC staining, the viable pollen was stained red (Figure 2C), while for the benzidine-H_2_O_2_ staining, the viable pollen was stained purple (Figure 2E). For the FDA staining, the viable pollen was stained green under a fluorescence microscope (Figure 2I).

The single linear regression analysis was performed on the results of the staining method and the results of the pollen germination in vitro. The correlation analysis between the FDA staining method and the pollen germination in vitro showed a high value (R^2^ = 0.705, *p* < 0.001; Figure S3). In contrast, MTT staining method showed a low correlation with germination in vitro (R^2^ = 0.239, *p* = 0.064; Figure S3). Although the above methods could distinguish viable and non-viable pollen, the FDA staining method proved to be more suitable for assessing the viability of *P. ostii* pollen, in comparison to the other three staining methods.

The FDA staining method is widely used in the determination of pollen viability [21]. Fluorescein diacetate (FDA) is a fluorescent dye that can enter living cells through the cell membrane and can be degraded by esterase to produce yellow-green fluorescent substances.

However, acetic carmine, methylene blue, peroxide, and I_2_-KI could not distinguish viable pollen and non-viable pollen (Figure 2B,D,F,G). In addition, when pollen grains were treated with inorganic acids, no pollen tubes could be observed (Figure 2H).

### 2.4. Thawing and Rehydration of Pollen Germination after Cryopreservation

Low temperature is helpful for long-term storage of pollen grains [22]. In addition, thawing and rehydration are important steps to improve germination in vitro, especially for pollen grains stored at low temperature. The samples frozen at −80 °C for one month were thawed at 4 °C for 0, 30, 60, and 90 min. The results showed that the germination percentage significantly increased with the extension of thawing time without rehydration (Table 5). Rehydration after thawing could significantly increase the germination percentage compared with no hydration. At the same hydration time, the germination percentage of pollen grains after thawing for 30 min was 77.18%, which was not significantly different from that of thawing for 60 and 90 min. Our findings suggest that rehydration after thawing has a positive effect on the germination. Through comparative analysis, we found that the pollen stored at −80 °C was thawed at 4 °C for 30 min and rehydrated at 25 °C for 30 min, which was suitable for germination after low-temperature storage.

### 2.5. Pollen Viability after Preservation at Different Temperature

**Figure 3:**
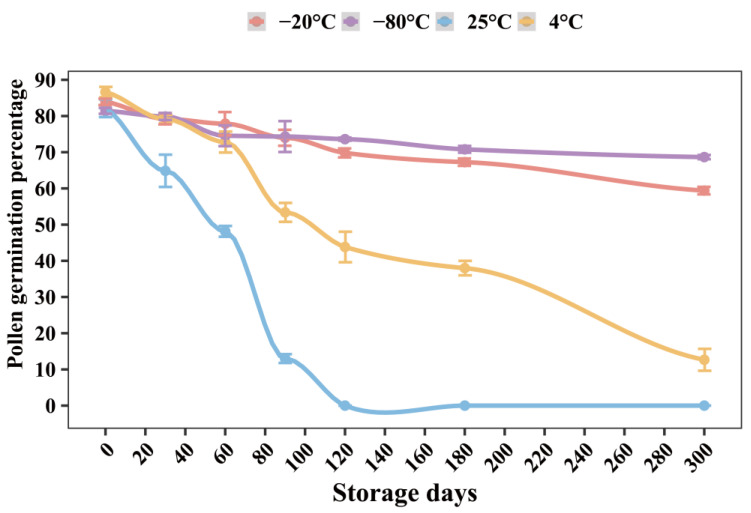
Pollen germination percentage (%) of *Paeonia ostii* at different preservation temperatures and times.

The germination percentage was analyzed in vitro, after different preservation temperatures (25, 4, −20, and −80 °C). The results showed that the germination percentage was only 45.64% after pollen storage at 25 °C for 60 days (Figure 3). With the increase of time, the viability of pollen grains at 25 °C gradually decreased. After having been stored at 25 °C for 180 days, the pollen grains would no longer germinate. Compared with fresh pollen grains, when they were stored at 4 °C for 60 days, the germination percentage decreased to 73.57%. Similarly, the germination percentage decreased rapidly to 13.5% at 4 °C for 300 days. Compared with other storage conditions, the germination percentage of pollen grains stored at −20 and −80 °C for 60 days was higher, 76.99% and 73.63%, respectively. Even when stored at −20 and −80 °C for 300 days, the germination percentage still reached 55.7% and 66.11%, respectively. Therefore, it was found that 4 °C was suitable for short-term storage of pollen grains, while −80 °C was suitable for long-term storage of pollen grains.

## 3. Discussion

Flowering plants produce seeds through double fertilization, which is a unique method of sexual reproduction among living organisms [23]. The normal development of pollen as the male gametophyte is crucial for successful fertilization during reproduction [24]. Previous studies showed that the pollen source and vitality could affect the seed setting rate [25] and the nutritional content of the seeds in cross-pollination [1,3]. In production, the flowering period of *P. ostii* is inconsistent, and understanding the pollen viability of *P. ostii* in vitro is important for improving the breeding efficiency through hybridization. It is well known that germination in vitro is one of the most effective ways to detect pollen viability in plants [26]. Among them, BK medium has been successfully used in pollen of some ornamental and forestry agricultural species. However, the germination percentage was different in BK medium [27,28]. Plants require specialized pollen germination media, suggesting that different components in the media can affect germination in vitro [29,30,31].

In pollen germination assays, sucrose is commonly used as an energy source for pollen, stimulating their germination and pollen tube growth [32]. In addition to sucrose, boron (B) is an essential trace element for plant species, and boron deficiency can inhibit pollen grains germination and lead to delayed growth of pollen tubes [33,34]. Boric acid is also an important substance for germination in vitro, and a low concentration of boric acid promotes germination and pollen tube elongation. Polyethylene glycol (PEG), an osmotic regulator not metabolized in pollen, is thought to regulate plasma membrane permeability [35]. The addition of PEG to the germination medium increases germination percentage and pollen tube elongation by preventing pollen tube bursting [36]. In this study, we found that sucrose, boric acid, and PEG had effects on the germination of *P. ostii* pollen grains, and the best combination was: 50 g/L sucrose, 100 mg/L boric acid, 50 g/L PEG.

In addition to these factors, the pH of the medium and the temperature are important factors affecting the germination percentage and the growth of pollen tubes [37]. In Hydrangea macrophylla, the optimum pH of the germination medium was 5.5–6.0 [38]. In the study, it was also found that the *P. ostii* pollen grains were easily affected by the pH and had a higher germination percentage under acidic conditions (pH = 5.4).

Furthermore, temperature is also an important regulator in germination medium. The sexual reproduction of plants is more sensitive to temperature than the vegetative process; high and low temperatures will affect the reproductive organs of plants [39]. The optimum germination temperature of hazelnut pollen was 20–25 °C [40]. On the other hand, low and high temperatures caused the accumulation of callose in pollen tubes to affect pollen germination and elongation, which was reported in other plant species [39]. Therefore, the germination of pollen grains requires a suitable temperature. In this experiment, it was found that the germination percentage of *P. ostii* pollen grains reached 85.05% when they were germinated at 25 °C for 120 min.

Various staining methods are also used to assess pollen viability [41]. These methods are characterized by intuitiveness and rapidity, and depend mainly on cell integrity, enzyme activity, and nutritional components [35]. Pollen viability of different plants can be assessed with specific staining methods [42]. Currently, there are very limited comparisons of staining methods for pollen, especially in the assessment of *P. ostii* pollen. Therefore, in this study, nine staining methods (MTT, acetic carmine, TTC, I_2_-KI, benzidine-H_2_O_2_, peroxide, methylene blue, inorganic acid, and FDA) were used to assess pollen viability of this species. MTT, TTC, benzidine-H_2_O_2_, and FDA could distinguish between viable and non-viable pollen. MTT, TTC, and benzidine-H_2_O_2_ staining methods are based on the activity of enzymes in pollen. Among them, benzidine-H_2_O_2_ stained viable pollen of *P. ostii* in a short period of time (5 min), which is mainly based on the peroxidase activity in pollen [43]. MTT and TTC staining methods are used to assess pollen viability according to the activity of dehydrogenase and reductase, respectively [44]. However, the FDA staining method assessed the viability of pollen by the intact plasma membrane and enzyme activity. Compared with other methods, the FDA staining method is widely used in the determination of pollen viability, due to its accuracy [14]. In this study, the results of the FDA staining method were more accurate compared with pollen germination in vitro, which indicated that FDA was suitable for measuring viability of *P. ostii* pollen grains.

Thawing and rehydration are important steps to increase the germination percentage of pollen in vitro, especially for low-temperature storage [45]. Low-temperature storage, which delays metabolism and physiological processes, is one of the long-term, effective, and safe pollen storage methods [46]. Therefore, prolonging pollen longevity and storing pollen in a suitable way are very important for the preservation of germplasm resources. In this study, it was found that the pollen grains stored at 25 °C lost their vitality and could not germinate after 120 days, and the germination percentage of pollen grains stored at 4 °C was reduced to half after 120 days. This result indicated that 4 °C was suitable only for short-term storage of pollen. On the other hand, the germination percentage of pollen grains stored at −20 °C and −80 °C for 300 days was 55.71% and 66.11%, respectively. This result indicated that −20 °C or −80 °C were suitable for long-term preservation of pollen grains. In the experiment, the germination percentage (58.15%) was lower after low-temperature storage without thawing and rehydration. When the pollen grains stored at low temperature were thawed for 30 min and rehydrated for 30 min, the germination percentage was 77.28%. Our study showed that germination percentage after low-temperature storage was enhanced by thawing and rehydration.

## 4. Materials and Methods

### 4.1. Plant Material and Pollen Grain Collection

Tree peony (*P. ostii*) was cultivated at Northwest A&F University (Yangling, Shaanxi, China). The stamens of *P. ostii* were collected before blooming on 9 April 2022. In order to collect pollen grains, the stamens were placed in a sulfuric acid paper and placed at 25 °C for 48 h, and the cracked stamens were passed through a 100-mesh sieve to collect pollen. After the pollen grains were dried in the shade at 25 °C for 2 h, they were put into a 2 mL cryopreservation tube.

### 4.2. Pollen Germination Medium

The germination medium was optimized using the pollen grains of *P. ostii*, referring to the method of BK medium and making appropriate modifications. In this study, one factor was changed while the other factors were kept constant at a time (single-factor experiment method) to investigate the effects of different substance concentrations in the medium (Table 6 and Tables S1–S7). The pH of the medium was adjusted with 1 N NaOH/1 N HCl [9]. Based on single-factor experiment method, four factors (sucrose, boric acid, PEG, pH) were selected, which have a greater impact on pollen grains, and the optimal combination of germination medium was screened using an orthogonal assay test strategy (OATS). The Petri dishes were cultured in a 25 °C incubator for 2 h, and five Petri dishes were prepared for each combination. The Olympus BX-53 microscope was used for pollen grains observation (no less than 100 pollen grains) and calculation of germination percentage. The pollen grains were considered germinated when the pollen tube length was twice the pollen grain diameter. The OATS is represented by the formula L_N_(V^K^), where N represents the number of combinations, V represents the maximum number of values that can be taken on any single-factor (levels), and K represents the number of factors tested. In general, a total of 9 combinations (3 levels with 5 replicates) including 4 germination medium components (L_9_(3^4^)) were used for orthogonal data analysis in this experiment (Table 2).

### 4.3. Pollen Germination Temperature

Once the optimal germination medium was identified, pollen grains were plated on the medium at five temperatures (15, 20, 25, 30, and 35 °C) and 85% humidity, and each treatment was replicated three times. For each treatment, the Petri dishes were observed for germination at 30, 60, 90, 120, and 180 min while not less than 100 pollen grains were observed. The pollen tube lengths were measured at different culture times under each temperature condition for 50 pollen tubes.

### 4.4. Pollen Staining

Nine different staining methods were used for *P.ostii* pollen grains. First, the staining method was performed according to its reference (Table 7). Second, the pollen grains were placed in the staining solution and stained at different temperatures for 2–30 min. Finally, 30 μL of the mixed solution of pollen grains and staining solution was placed on a glass slide, and a cover glass was attached for observation with an Olympus BX-53 microscope. There were three replicates for each method and five slides per accession.

### 4.5. Thawing and Rehydration of Pollen after Cryopreservation and In Vitro Germination

The pollen grains stored at −80 °C for 30 days were taken out and placed at 4 °C for 0, 30, 60, and 90 min to thaw, then placed in a water bath at 25 °C for rehydration for 0, 30, and 60 min. The hydrated pollen grains were placed in the medium, cultured at 25 °C for 2 h, and then the pollen grains were observed under an Olympus BX-53 microscope.

### 4.6. Pollen Storage Conditions

The pollen grains were stored at four different temperatures (25, 4, −20, −80 °C) for 300 days. The pollen grains were taken out at 30, 60, 90, 120, 180, and 300 days, respectively, thawed at 4 °C for 30 min, and hydrated in a water bath at 25 °C for 30 min. In order to ensure the consistency of the experiment, the pollen grains stored at 25 °C, 4 °C, and −20 °C were also subjected to the above process. Afterwards, the pollen grains were placed in the germination medium, germinated at 25 °C for 2 h, and the germination percentage was calculated.

### 4.7. Statistical Analysis

Data analyses were analyzed with SPSS Statistics 17.0 (SPSS Inc., Chicago, IL, USA). The data were analyzed by calculating means ± SD. To assess the normality and homogeneity of the datasets, Shapiro–Wilk’s test and Levene’s test were employed, respectively. A one-way ANOVA was conducted followed by Tukey’s test and different letters were used to indicate significant differences by Tukey’s test (*p* < 0.05). The OATS was used to determine the factors and levels for the composition of pollen germination media. Furthermore, a single linear regression analysis was performed to quantify the relationships between the pollen viability estimates obtained through staining and the in vitro pollen germination percentages achieved using the optimal media compositions. The pollen germination percentage (%) and pollen tube length (μm) were counted using ImageJ 1.46r software. The ggplot2/R package was used for visualization [53].

## 5. Conclusions

The germination of the *P. ostii* pollen grain are affected by sucrose, PEG, pH, and boric acid. In this study, the optimal medium containing 50 g/L sucrose, 100 mg/L boric acid, 50 g/L PEG, 100 mg/L potassium nitrate, 300 mg/L calcium nitrate, and 200 mg/L magnesium sulfate at pH 5.4 was suggested for the germination of *P. ostii* pollen grains in vitro. By comparing the germination temperature and time, it was found that germination at 25 °C for 2 h was the suitable condition for *P. ostii* pollen grains. Among the nine viability staining methods (MTT, acetic carmine, TTC, I_2_-KI, benzidine-H_2_O_2_, peroxide, methylene blue, inorganic acid, and FDA), MTT, TTC, benzidine-H_2_O_2_, and FDA were able to distinguish viable pollen from non-viable pollen. The result of the FDA staining method was similar to the germination percentage in vitro, which was used for *P. ostii* pollen grains. In addition, this study found that thawing pollen stored at low temperature for 30 min at 4 °C and rehydrating for 30 min at 25 °C increased the germination percentage of *P. ostii* pollen grains in vitro. A subsequent study showed that *P. ostii* pollen grains were suitable for short-term storage at 4 °C, and long-term storage at −80 °C facilitated pollination in the next growing season.

## Figures and Tables

**Table 1:** The composition of *Paeonia ostii* pollen grains germination medium by orthogonal assay test strategy (OATS).

Levels	Sucrose (S) (g/L)	Boric Acid (B) (mg/L)	PEG6000 (P) (g/L)	pH (H)
1	50	50	50	5.4
2	80	80	100	5.8
3	100	100	150	6.2

**Table 2:** The analysis of the effect of different media on the pollen grains of *Paeonia ostii* using an orthogonal assay test strategy (OATS).

Code	Factors					Germination Percentage (%)					
	Sucrose	Boric Acid	PEG6000	pH	Treatment	Replicate I	Replicate II	Replicate III	Replicate IV	Replicate V	Average
	S	B	P	H	T						
1	**S1**	B1	**P1**	**H1**	S1B1P1H1	84.52	84.74	82.83	84.05	82.02	83.63
2	S1	B2	P2	H2	S1B2P2H2	80.11	82.86	80	79	80.95	80.58
3	S1	**B3**	P3	H3	S1B3P3H3	45	58.57	51.08	50.15	51.19	51.2
4	S2	B1	P2	H3	S2B1P2H3	48.92	48.15	39.33	48.82	47.95	46.63
5	S2	B2	P3	H1	S2B2P3H1	88.24	79.25	85.96	70.39	85.71	81.91
6	S2	B3	P1	H2	S2B3P1H2	80.75	84.74	88.24	82.14	81.18	83.41
7	S3	B1	P3	H2	S3B1P3H2	68.75	75.76	68.33	60	79.03	70.37
8	S3	B2	P1	H3	S3B2P1H3	48.53	47.5	47.89	48.21	47.27	47.88
9	S3	B3	P2	H1	S3B3P2H1	82.93	82.97	85.35	82.36	82.31	83.18
K1	**1077.07**	1003.2	**1074.61**	**1243.63**							
K2	1059.77	1051.87	1052.01	1171.84							
K3	1007.19	**1088.96**	1017.41	728.56							
x1	**71.8**	66.88	**71.64**	**82.91**							
x2	70.65	70.12	70.13	78.12							
x3	67.14	**72.6**	67.83	48.57							
R	4.66	5.72	1.51	34.34							

Ki represents the sum of the different levels for the *i*th factor, xi denotes the mean of the different levels for the ith factor, and R represents the difference in range between the maximum and minimum values. In addition, three levels of sucrose (S), boric acid (B), PEG6000 (P), pH (H), were shown in Table 1. Bold numbers indicate the highest values, while bold letters represent the optimal components.

**Table 3:** The effects of different temperatures on germination percentage (%) of *Paeonia ostii* pollen grains (*p* < 0.05).

Time (min)	Culture Temperature (°C)				
	15	20	25	30	35
30	1.7 ± 1.7 d	11.19 ± 0.51 d	13.67 ± 0.51 d	6.33 ± 2.64 c	0 ± 0 d
60	16.33 ± 0.34 c	39.83 ± 1.69 c	37.69 ± 1.92 c	26.67 ± 0.33 b	9.09 ± 0.31 c
90	23.18 ± 0.49 b	58.42 ± 1.16 b	57.82 ± 0.54 b	50.54 ± 0.64 b	14.08 ± 1.01 b
120	52.22 ± 4.19 a	73.67 ± 0.87 a	85.05 ± 0.58 a	66.01 ± 1.83 a	24.23 ± 1.21 a
180	51.04 ± 2.3 a	74.36 ± 1.29 a	84.34 ± 1.33 a	65.31 ± 1.3 a	25.2 ± 0.71 a

The letter shows difference at a significant level in the column.

**Table 4:** The effects of different temperatures on pollen tube length (μm) of *Paeonia ostii* pollen grains (*p* < 0.05).

Time (min)	Culture Temperature (°C)				
	15	20	25	30	35
30	2.54 ± 1.26 d	46.12 ± 1.33 e	55.69 ± 1.25 e	67.74 ± 2.03 e	0 ± 0 e
60	41.73 ± 0.34 c	88.38 ± 1.91 d	100.57 ± 2.71 d	107.36 ± 2.35 d	39.11 ± 0.89 d
90	96.82 ± 2.22 b	123.01 ± 2.31 c	149.16 ± 2.63 c	145.16 ± 3.02 c	79.79 ± 2.89 c
120	135.73 ± 4.57 a	156.14 ± 4.21 b	245.06 ± 5.86 b	251.51 ± 7.71 b	123.67 ± 3.09 b
180	248.24 ± 7.5 a	284.63 ± 11.87 a	390.76 ± 5.63 a	331.52 ± 1.26 a	177.47 ± 7.47 a

The letter shows difference at a significant level in the column.

**Table 5:** The effect of different unfreezing and rehydration times on pollen grains of *Paeonia ostii* (*p* < 0.05).

Unfreezing Time at 4 °C (min)	Rehydration Time at 25 °C (min)	Germination Percentage (%)
0	0	58.15 ± 0.4 c
30	0	58.94 ± 1.51 c
60	0	65.68 ± 0.89 b
90	0	66.94 ± 0.92 b
30	30	77.18 ± 0.86 a
60	30	77.25 ± 0.38 a
90	30	74.34 ± 0.86 a
30	60	76.68 ± 0.66 a
60	60	76.97 ± 0.47 a
90	60	77.76 ± 0.82 a

The letter shows difference at a significant level in the column.

**Table 6:** The composition of *Paeonia ostii* pollen grains germination medium by single-factor experiment.

Levels	Sucrose (S) (g/L)	Boric Acid (B) (mg/L)	Calcium Nitrate (Ca) (mg/L)	Magnesium Sulfate (Mg) (mg/L)	Potassium Nitrate (K) (mg/L)	PEG6000 (P) (g/L)	pH (H)
1	200	300	500	500	300	250	5.4
2	150	200	400	400	200	200	5.8
3	100	100	300	300	100	150	6.2
4	80	80	200	200	80	100	7.5
5	50	50	100	100	50	50	8.4
6	0	0	0	0	0	0	9.6

**Table 7:** Methods of staining solution and staining condition.

Method	Staining Solution	Staining Conditions	
		Time (min)	Temperature (°C)
Acetic carmine	1% Acetic carmine [47]	5	25
Benzidine-H_2_O_2_	0.5% benzidine and 0.3% H_2_O_2_ [48]	5	25
I_2_-KI	0.5% I_2_-KI [49]	5	25
Inorganic acid	14.4% H_2_SO_4_ [50]	5	25
Methylene blue	1% methylene blue [5]	2	25
MTT	0.3% MTT: 0.3 g MTT dissolved in 100 mL PBS [9]	30	35
Peroxide	A solution: 0.2 g of benzidine was dissolved in 100 mL of 50% alcohol, 0.15 g of naphthol was dissolved in 100 mL of 50% alcohol, 0.25 g of sodium carbonate was dissolved in 100 mL of distilled water, and a mixed solution was prepared in equal amountsB solution: 0.3% H_2_O_2_ Take solution A and solution B in equal volumes during the test [51]	30	35
TTC	0.5% TTC: 0.5 g TTC dissolved in 100 mL 95% alcohol [52]	30	35
Fluorescein diacetate (FDA)	FDA solution: 25 μg FDA dissolved in 1 mL acetone [8]	20	25

## Data Availability

The data is contained within the article and Supplementary Materials.

## Supplementary Materials

The following supporting information can be downloaded at: https://www.mdpi.com/article/10.3390/plants12132460/s1, Figure S1: The effects of different concentrations of calcium nitrate, magnesium sulfate, and potassium nitrate on pollen germination percentage and pollen tube length of *Paeonia ostii*; Figure S2: The pollen tube growth and pollen germination of *Paeonia ostii* using an orthogonal assay test strategy; Figure S3: The single linear regression of in vitro germinated pollen (%) relative to stained pollen (%); Tables S1–S7: Concentration of each component in single-factor experiment.

## References

[1] Zhang Q., Yu R., Sun D., Rahman M.M., Xie L., Hu J., He L., Kilaru A., Niu L., Zhang Y. (2019). Comparative Transcriptome Analysis Reveals an Efficient Mechanism for α-Linolenic Acid Synthesis in Tree Peony Seeds. Int. J. Mol. Sci..

[2] Zhang Q.-Y., Yu R., Xie L.-H., Rahman M.M., Kilaru A., Niu L.-X., Zhang Y.-L. (2018). Fatty Acid and Associated Gene Expression Analyses of Three Tree Peony Species Reveal Key Genes for α-Linolenic Acid Synthesis in Seeds. Front. Plant Sci..

[3] Xie L., Hu J., Zhang Q., Sun Q., Zhang Y., Niu L. (2019). Influence of Pollen Sources on the Expression of FA and TAG Biosynthetic Pathway Genes in Seeds of Paeonia Rockii during the Rapid Oil Accumulation. Sci. Hortic..

[4] Zhang K., He C., Wang S., Hou X. (2022). Influence of Pollination Methods on Fruit Development, Fruit Yield and Oil Quality in Oil Tree Peony. Sci. Hortic..

[5] Kumar A., Chowdhury R.K., Dahiya O.S. (1995). Pollen Viability and Stigma Receptivity in Relation to Meteorological Parameters in Pearl Millet. Seed Sci. Technol..

[6] Mazzeo A., Palasciano M., Gallotta A., Camposeo S., Pacifico A., Ferrara G. (2014). Amount and Quality of Pollen Grains in Four Olive (*Olea europaea* L.) Cultivars as Affected by ‘on’ and ‘off’ Years. Sci. Hortic..

[7] Dickinson D.B. (1968). Rapid Starch Synthesis Associated with Increased Respiration in Germinating Lily Pollen 1. Plant Physiol..

[8] Zhou H., Yin H., Chen J., Liu X., Gao Y., Wu J., Zhang S. (2016). Gene-Expression Profile of Developing Pollen Tube of Pyrus Bretschneideri. Gene Expr. Patterns.

[9] Kumari M., Prasad A., ur Rahman L., Mathur A.K., Mathur A. (2022). In Vitro Germination, Storage and Microscopic Studies of Pollen Grains of Four Ocimum Species. Ind. Crops Prod..

[10] Zhang D., Wengier D., Shuai B., Gui C.-P., Muschietti J., McCormick S., Tang W.-H. (2008). The Pollen Receptor Kinase LePRK2 Mediates Growth-Promoting Signals and Positively Regulates Pollen Germination and Tube Growth. Plant Physiol..

[11] Lora J., de Oteyza M.A.P., Fuentetaja P., Hormaza J.I. (2006). Low Temperature Storage and in Vitro Germination of Cherimoya (Annona Cherimola Mill.) Pollen. Sci. Hortic..

[12] Karimi H.R., Zeraatkar H. (2016). Effects of Artificial Pollination Using Pollen Suspension Spray on Nut and Kernel Quality of Pistachio Cultivar Owhadi. Int. J. Fruit Sci..

[13] Ćalić D., Milojević J., Belić M., Miletić R., Zdravković-Korać S. (2021). Impact of Storage Temperature on Pollen Viability and Germinability of Four Serbian Autochthon Apple Cultivars. Front. Plant Sci..

[14] Buitink J., Claessens M.M.A.E., Hemminga M.A., Hoekstra F.A. (1998). Influence of Water Content and Temperature on Molecular Mobility and Intracellular Glasses in Seeds and Pollen1. Plant Physiol..

[15] Machado C.D.A., Moura C.R.F., de Lemos E.E.P., Ramos S.R.R., Ribeiro F.E., Lédo A.D.S. (2014). Pollen Grain Viability of Coconut Accessions at Low Temperatures. Acta Sci. Agron..

[16] Zambon C.R., Silva L.F.D.O.D., Pio R., de Figueiredo M.A., Silva K.N. (2014). Establishment of Growth Medium and Quantification of Germination of Pollen Grains of Quince Tree Cultivars. Rev. Bras. Frutic..

[17] Boavida L.C., McCormick S. (2007). Technical Advance: Temperature as a Determinant Factor for Increased and Reproducible in Vitro Pollen Germination in Arabidopsis Thaliana. Plant J..

[18] Hamilton E.S., Haswell E.S. (2017). The Tension-Sensitive Ion Transport Activity of MSL8 Is Critical for Its Function in Pollen Hydration and Germination. Plant Cell Physiol..

[19] Jayaprakash P., Sarla N. (2001). Development of an Improved Medium for Germination of *Cajanus cajan* (L.) Millsp. Pollen in Vitro. J. Exp. Bot..

[20] Bou Daher F., Chebli Y., Geitmann A. (2009). Optimization of Conditions for Germination of Cold-Stored Arabidopsis Thaliana Pollen. Plant Cell Rep..

[21] Podolyan A., Maksimov N., Breygina M. (2019). Redox-Regulation of Ion Homeostasis in Growing Lily Pollen Tubes. J. Plant Physiol..

[22] Mesnoua M., Roumani M., Salem A. (2018). The Effect of Pollen Storage Temperatures on Pollen Viability, Fruit Set and Fruit Quality of Six Date Palm Cultivars. Sci. Hortic..

[23] Takahashi T., Mori T., Ueda K., Yamada L., Nagahara S., Higashiyama T., Sawada H., Igawa T. (2018). The Male Gamete Membrane Protein DMP9/DAU2 Is Required for Double Fertilization in Flowering Plants. Development.

[24] Liang M., Cao Z., Zhu A., Liu Y., Tao M., Yang H., Xu Q., Wang S., Liu J., Li Y. (2020). Evolution of Self-Compatibility by a Mutant Sm-RNase in Citrus. Nat. Plants.

[25] Fayos O., Echávarri B., Vallés M.P., Mallor C., Garcés-Claver A., Castillo A.M. (2022). A Simple and Efficient Method for Onion Pollen Preservation: Germination, Dehydration, Storage Conditions, and Seed Production. Sci. Hortic..

[26] Daniel I.O. (2011). Exploring Storage Protocols for Yam (*Dioscorea* spp.) Pollen Genebanking. Afr. J. Biotechnol..

[27] Sousa A.S., Santos M.G.M., Pelacani C.R., Santos F.d.A.R. (2016). Testing Culture Media for Pollen Germination of Elaeis Guineensis Jacq. (Oil Palm, Arecaceae). Bot. J. Linn. Soc..

[28] dos Santos Sousa A., Rego E.J.L., Santos F.d.A.R.d. (2013). Viability and Action of CPL Lectin on in Vitro Germinability of Pollen Grains of Malpighia Emarginata DC.—(Malpighiaceae). Am. J. Plant Sci..

[29] Fang K., Xie P., Zhang Q., Xing Y., Cao Q., Qin L. (2020). Aluminum Toxicity-Induced Pollen Tube Growth Inhibition in Apple (Malus Domestica) Is Mediated by Interrupting Calcium Dynamics and Modification of Cell Wall Components. Environ. Exp. Bot..

[30] Kuroki K., Takemura Y., Mingfeng J., Marumori H., Teratani N., Matsumoto K., Matsumoto T., Tamura F. (2017). Pear Pollen Selection Using Higher Germination Properties at Low Temperatures and the Effect on the Fruit Set and Quality of Japanese Pear Cultivars. Sci. Hortic..

[31] Wood B.W. (2017). Flavonoids, Alkali Earth, and Rare Earth Elements Affect Pecan Pollen Germination. HortScience.

[32] Okusaka K., Hiratsuka S. (2009). Fructose Inhibits Pear Pollen Germination on Agar Medium without Loss of Viability. Sci. Hortic..

[33] Fang K.F., Du B.S., Zhang Q., Xing Y., Cao Q.Q., Qin L. (2019). Boron Deficiency Alters Cytosolic Ca^2+^ Concentration and Affects the Cell Wall Components of Pollen Tubes in Malus Domestica. Plant Biol..

[34] Wang Q., Lu L., Wu X., Li Y., Lin J. (2003). Boron Influences Pollen Germination and Pollen Tube Growth in Picea Meyeri. Tree Physiol..

[35] Shivanna K.R., Sawhney V.K. (1995). Polyethylene Glycol Improves the in Vitro Growth of Brassica Pollen Tubes without Loss in Germination. J. Exp. Bot..

[36] Sakhanokho H.F., Rajasekaran K. (2010). Pollen Biology of Ornamental Ginger (*Hedychium* spp. J. Koenig). Sci. Hortic..

[37] Cameron C., Geitmann A. (2018). Cell Mechanics of Pollen Tube Growth. Curr. Opin. Genet. Dev..

[38] Alexander L.W. (2019). Optimizing Pollen Germination and Pollen Viability Estimates for Hydrangea Macrophylla, Dichroa Febrifuga, and Their Hybrids. Sci. Hortic..

[39] Hebbar K.B., Rose H.M., Nair A.R., Kannan S., Niral V., Arivalagan M., Gupta A., Samsudeen K., Chandran K.P., Chowdappa P. (2018). Differences in in Vitro Pollen Germination and Pollen Tube Growth of Coconut (*Cocos nucifera* L.) Cultivars in Response to High Temperature Stress. Environ. Exp. Bot..

[40] Çetinbaş-Genç A., Cai G., Vardar F., Ünal M. (2019). Differential Effects of Low and High Temperature Stress on Pollen Germination and Tube Length of Hazelnut (*Corylus avellana* L.) Genotypes. Sci. Hortic..

[41] Impe D., Reitz J., Köpnick C., Rolletschek H., Börner A., Senula A., Nagel M. (2020). Assessment of Pollen Viability for Wheat. Front. Plant Sci..

[42] Kosel J., Vižintin L., Majer A., Bohanec B. (2018). Staining for Viability Testing, Germination and Maturation of *Sambucus nigra* L. Pollen in Vitro. Biotech. Histochem..

[43] dos Santos K.S., Passos A.R., Serejo J.A.d.S., Lino L.S.M., Figueiredo M.C.C., Santos R.M.F. (2017). Microsporogenesis and Pollen Viability in Physalis Ixocarpa. Cytologia.

[44] Grela E., Kozłowska J., Grabowiecka A. (2018). Current Methodology of MTT Assay in Bacteria—A Review. Acta Histochem..

[45] Peng H.-Z., Jin Q.-Y., Ye H.-L., Zhu T.-J. (2015). A Novel in Vitro Germination Method Revealed the Influence of Environmental Variance on the Pecan Pollen Viability. Sci. Hortic..

[46] Polesi L.G., Goeten D., Fraga H.P.d.F., Steiner N., Guerra M.P. (2023). Enzymatic Antioxidant System Activation Assures the Viability of Guadua Chacoensis (Bambusoideae, Poaceae) Embryogenic Cultures during Cryopreservation. Plants.

[47] Ferrara G., Camposeo S., Palasciano M., Godini A. (2007). Production of Total and Stainable Pollen Grains in *Olea europaea* L. Grana.

[48] Koga Y., Akihama T., Fujimaki H., Yokoo M. (1971). Studies on the Longevity of Pollen Grains of Rice, *Oriza sativa* L. Cytologia.

[49] Du G., Xu J., Gao C., Lu J., Li Q., Du J., Lv M., Sun X. (2019). Effect of Low Storage Temperature on Pollen Viability of Fifteen Herbaceous Peonies. Biotechnol. Rep..

[50] van Gelderen E., Fossey A., Robbertse P.J. (1995). The Criteria of Measurement of the Inorganic Acid Test of Pollen Viability. S. Afr. J. Bot..

[51] Mukherjee S., Datta A.K. (2013). Induced Genetic Male Sterility in *Nigella sativa* L. (Black Cumin). Cytologia.

[52] Luo S., Zhang K., Zhong W.-P., Chen P., Fan X.-M., Yuan D.-Y. (2020). Optimization of in Vitro Pollen Germination and Pollen Viability Tests for Castanea Mollissima and Castanea Henryi. Sci. Hortic..

[53] Cran—Package Survival. https://cran.r-project.org/web/packages/survival/index.html.

