# Supplementary material for: Optimization of In Vitro Germination, Viability Tests and Storage of Paeonia ostii Pollen
Source: Plants (Basel). 2023 Jun 27;12(13):2460. doi: 10.3390/plants12132460 (PMC10346979; doi:10.3390/plants12132460)
Supplement: Supplementary file 1 [file plants-12-02460-s001.zip › plants-2402934-supplementary.pdf]

plants

Supplementary Materials

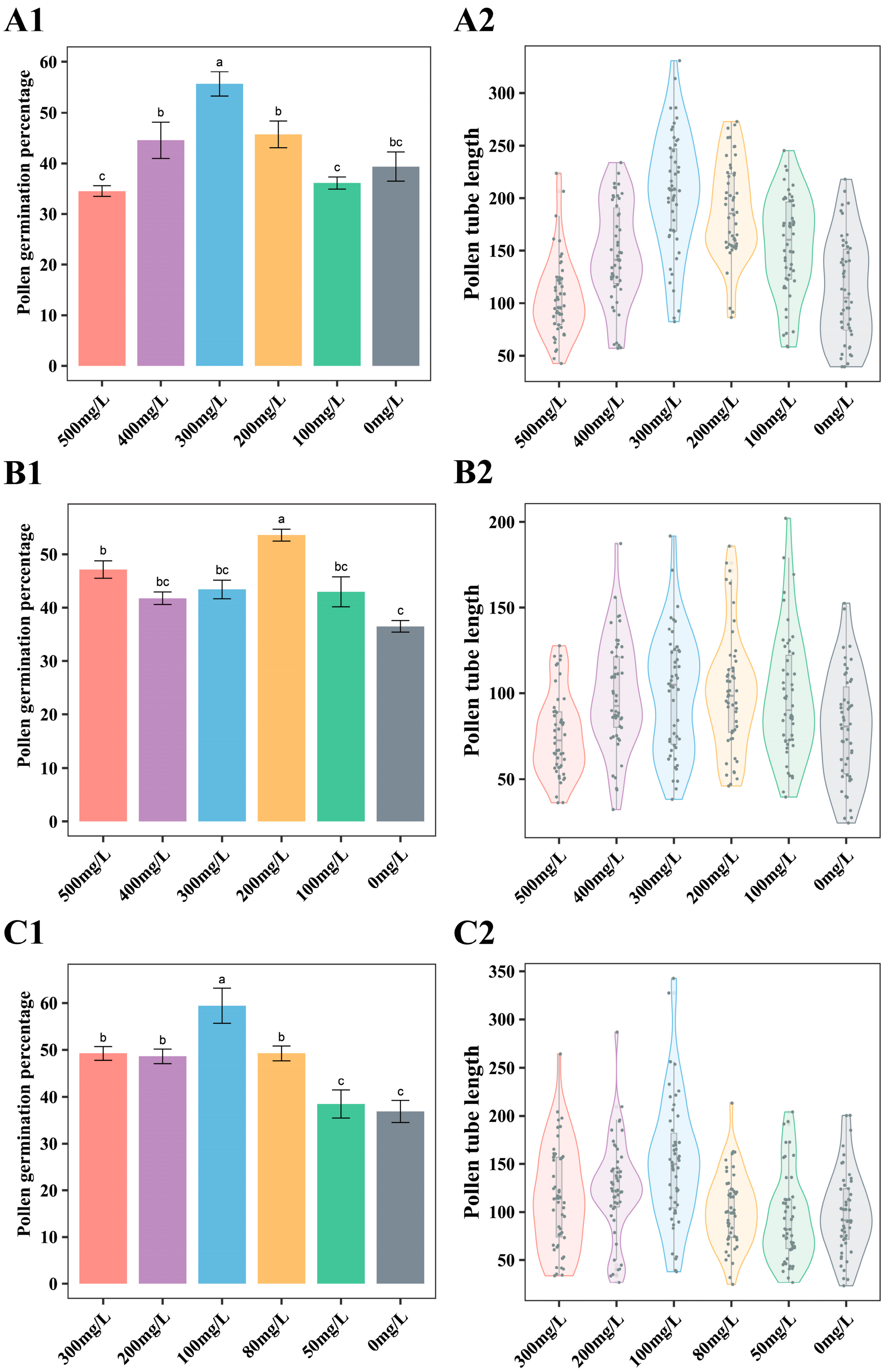

Figure S1. Pollen germination percentage (%) and pollen tube length (µm) of *Paeonia ostii* at different concentrations (A1-A2): Calcium nitrate; (B1-B2): Magnesium sulfate; (C1-C2): Potassium nitrate.

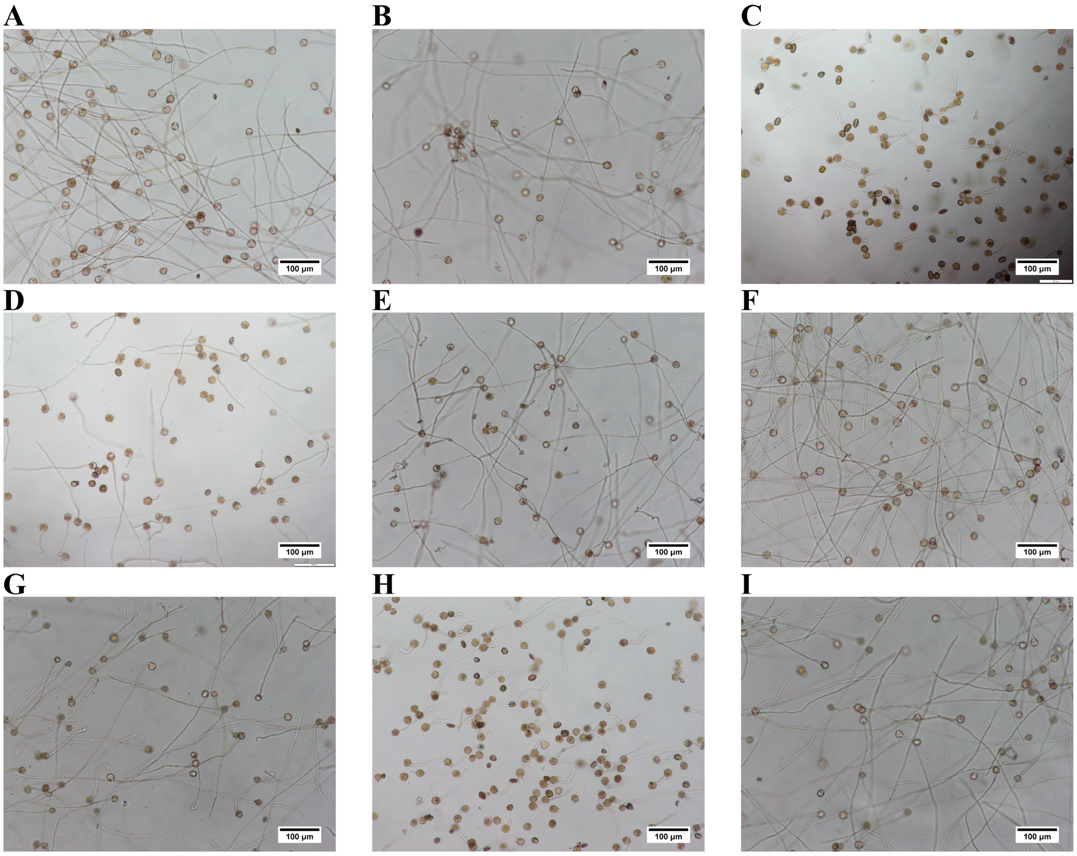

Figure S2. The pollen tube growth and pollen germination of *Paeonia ostii* in different mediums using an orthogonal assay test strategy (A-G represents 1-9 in Table 4). Bar=100um.

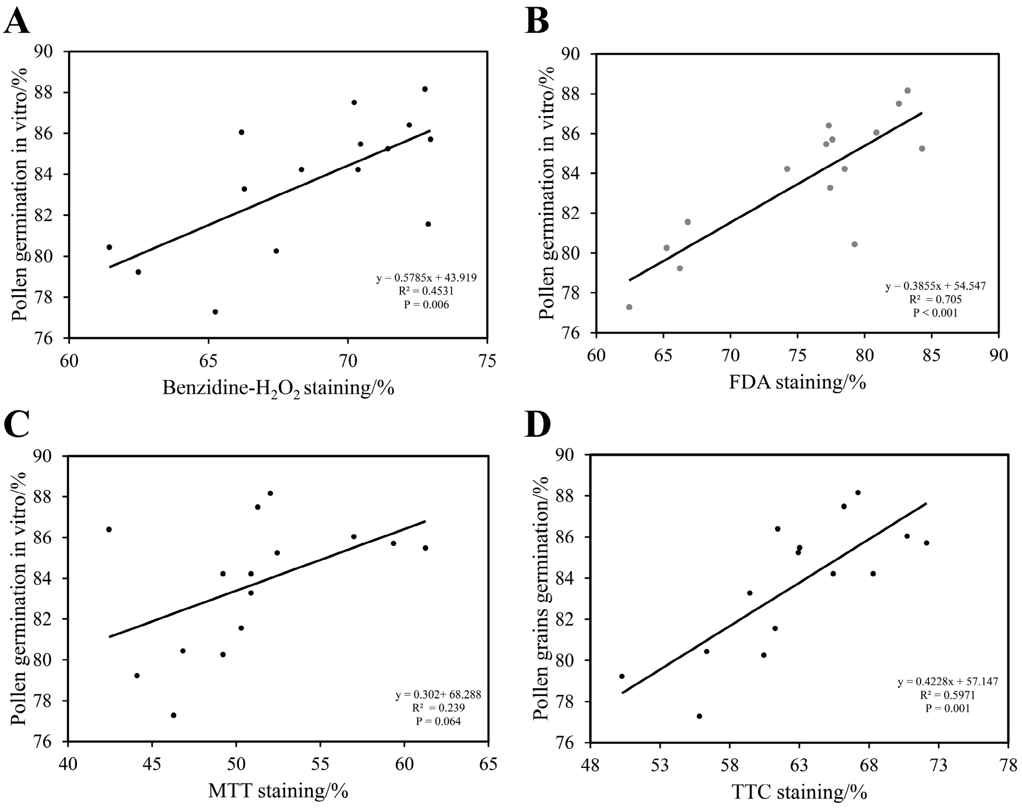

Figure S3. Single linear regression of pollen germination in vitro (%) relative to different pollen staining method (%) for *Paeonia ostii*. Each point represents the value of one replicate.

Table S1. Concentration of each component in single factor experiment with sucrose.

Levels	Sucrose (S) (g/L)	Boric acid (B) (mg/L)	Calcium nitrate (Ca) (mg/L)	Magnesium sulfate (Mg) (mg/L)	Potassium nitrate (K) (mg/L)	PEG6000 (P) (g/L)	pH (H)
1	200	100	300	200	100	150	6.2
2	150	100	300	200	100	150	6.2
3	100	100	300	200	100	150	6.2
4	80	100	300	200	100	150	6.2
5	50	100	300	200	100	150	6.2
6	0	100	300	200	100	150	6.2

Table S2. Concentration of each component in single factor experiment with boric acid.

Levels	Sucrose (S) (g/L)	Boric acid (B) (mg/L)	Calcium nitrate (Ca) (mg/L)	Magnesium sulfate (Mg) (mg/L)	Potassium nitrate (K) (mg/L)	PEG6000 (P) (g/L)	pH (H)
1	100	300	300	200	100	150	6.2
2	100	200	300	200	100	150	6.2
3	100	100	300	200	100	150	6.2
4	100	80	300	200	100	150	6.2
5	100	50	300	200	100	150	6.2
6	100	0	300	200	100	150	6.2

Table S3. Concentration of each component in single factor experiment with calcium nitrate.

Levels	Sucrose (S) (g/L)	Boric acid (B) (mg/L)	Calcium nitrate (Ca) (mg/L)	Magnesium sulfate (Mg) (mg/L)	Potassium nitrate (K) (mg/L)	PEG6000 (P) (g/L)	pH (H)
1	100	100	500	200	100	150	6.2
2	100	100	400	200	100	150	6.2
3	100	100	300	200	100	150	6.2
4	100	100	200	200	100	150	6.2
5	100	100	100	200	100	150	6.2
6	100	100	0	200	100	150	6.2

Table S4. Concentration of each component in single factor experiment with magnesium sulfate.

Levels	Sucrose (S) (g/L)	Boric acid (B) (mg/L)	Calcium nitrate (Ca) (mg/L)	Magnesium sulfate (Mg) (mg/L)	Potassium nitrate (K) (mg/L)	PEG6000 (P) (g/L)	pH (H)
1	100	100	300	500	100	150	6.2
2	100	100	300	400	100	150	6.2
3	100	100	300	300	100	150	6.2
4	100	100	300	200	100	150	6.2
5	100	100	300	100	100	150	6.2
6	100	100	300	0	100	150	6.2

Table S5. Concentration of each component in single factor experiment with potassium ni-trate.

Levels	Sucrose (S) (g/L)	Boric acid (B) (mg/L)	Calcium nitrate (Ca) (mg/L)	Magnesium sul-fate (Mg) (mg/L)	Potassium ni-trate (K) (mg/L)	PEG6000 (P) (g/L)	pH (H)
1					300		
2					200		
3	100	100	300		100	150	6.2
4				200	80		
5					50		
6					0		

Table S6. Concentration of each component in single factor experiment with PEG6000.

Levels	Sucrose (S) (g/L)	Boric acid (B) (mg/L)	Calcium nitrate (Ca) (mg/L)	Magnesium sul-fate (Mg) (mg/L)	Potassium ni-trate (K) (mg/L)	PEG6000 (P) (g/L)	pH (H)
1						250	
2						200	
3	100	100	300	200	100	150	6.2
4						100	
5						50	
6						0	

Table S7. Concentration of each component in single factor experiment with pH.

Levels	Sucrose (S) (g/L)	Boric acid (B) (mg/L)	Calcium nitrate (Ca) (mg/L)	Magnesium sul-fate (Mg) (mg/L)	Potassium ni-trate (K) (mg/L)	PEG6000 (P) (g/L)	pH (H)
1							5.4
2							5.8
3	100	100	300	200	100	150	6.2
4							7.5
5							8.4
6							9.6

Disclaimer/Publisher's Note: The statements, opinions and data contained in all publications are solely those of the individual author(s) and contributor(s) and not of MDPI and/or the editor(s). MDPI and/or the editor(s) disclaim responsibility for any injury to people or property resulting from any ideas, methods, instructions or products referred to in the content.
